# Intra-articular injection of orthobiologics in patients undergoing high tibial osteotomy for knee osteoarthritis is safe and effective – a systematic review

**DOI:** 10.1186/s40634-021-00387-2

**Published:** 2021-09-27

**Authors:** Brjan Kaiji Betzler, Aiman Haziq Bin Muhammad Ridzwan Chew, Hamid Rahmatullah Bin Abd Razak

**Affiliations:** 1 Lee Kong Chian School of Medicine, Nanyang Technological University, 59 Nanyang Drive, Experimental Medicine Building, Singapore, 636921 Singapore; 2 Adelaide Medical School, Faculty of Health and Medical Sciences, 30 Frome Rd, Adelaide, SA 5000 Australia; 3 Department of Orthopaedic Surgery, Sengkang General Hospital, 110 Sengkang East Way, Singapore, 544886 Singapore; 4 SingHealth Duke-NUS Musculoskeletal Sciences Academic Clinical Programme, 20 College Road, Academia Level 4, Singapore, 169865 Singapore

**Keywords:** Osteotomy, Cartilage repair, Knee, Biologics, Osteoarthritis

## Abstract

**Purpose:**

To qualitatively evaluate the current evidence reporting outcomes of intra-articular injection of orthobiologics in patients undergoing high tibial osteotomy (HTO) for osteoarthritis of the knee.

**Methods:**

A systematic search methodology of the PUBMED, EMBASE, and CINAHL databases was conducted in July 2021. The search workflow was in adherence to the Preferred Reporting Items for Systematic Reviews and Meta-Analyses (PRISMA). The following inclusion criteria were adopted: clinical trials of any level of evidence, reporting outcomes following intra-articular injection of orthobiologics during high tibial osteotomy for knee osteoarthritis, with a minimum number of 10 patients treated. Duplicate data, studies on implanted orthobiologics and articles not written in English were excluded from this review.

**Results:**

Eight studies were included in this review, with a total of 585 patients. Outcomes were discussed based on the types of orthobiologics used: (i) Platelet-Rich Plasma (PRP), (ii) Bone Marrow Aspirate Concentrate (BMAC), and (iii) Injected Mesenchymal Stem Cells (MSCs). Two studies utilised PRP, 4 studies utilised BMAC and 4 studies utilised injected MSCs.. Three studies provided Level II evidence and five studies provided Level III evidence. Statistically significant improvements in outcomes were documented in multiple trials, with few patients experiencing adverse events.

**Conclusion:**

Intra-articular injection of orthobiologics in patients undergoing HTO is safe and effective with good outcomes reported. Due to the lack of high-level evidence, further research is required before this can be considered standard of care.

**Level of evidence:**

III

## Introduction

Osteoarthritis (OA) is a degenerative bone disease characterised by loss of cartilage, bone remodelling in the adjacent bone structures, and inflammation of surrounding tissues [1]. Globally, it is the most prevalent degenerative joint disease [2], and the most common cause of knee pain. Deformities seen in knee OA such as genu varum further worsens function by altering the mechanical axis of the lower limb, placing additional stress on the arthritic medial compartment. Treatment modalities of OA to date have primarily focused on reducing the rate of cartilage degeneration. However, newer techniques have evolved, focusing on increasing the rate of cartilage regeneration.

High tibial osteotomy (HTO) is an effective procedure in the management of medial compartment knee OA with varus deformity, in young or physically active patients [3, 4]. It corrects the mechanical axis of the knee, reducing the rate of cartilage degeneration by improving weight distribution within the knee joint [5, 6]. Besides improved outcomes, several studies have also reported cartilage regeneration [7–10]. Concurrent procedures, such as the injection of orthobiologics during a HTO, have shown promise in enhancing cartilage regeneration in knee OA.

Orthobiologics are a relatively new treatment modality that has gained popularity recently due to its minimally invasive nature, and the potential for healing and recovery [11]. Broadly, orthobiologics include platelet rich plasma (PRP), plasma rich in growth factors (PRGF), bone marrow aspirate concentrate (BMAC) and mesenchymal stem cells (MSC). These products have the potential to aid in regeneration and recovery of cartilage [12]. While PRP and PRGF are rich in growth factors, BMAC and MSC both contain stem cells, with efficacy depending on multiple factors including source, proliferation capacity, and concentration of growth factors. It is important to note that PRP and BMAC are considered point of care treatment modalities, whereas MSCs typically require expansion prior to injection. Recent studies have reported on the efficacy of these orthobiologic agents. They have shown to enhance the quality of cartilage regeneration which in turn has contributed to better clinical outcomes following HTO [6, 10, 13–15].

Despite promising literature on the intra-articular injection of orthobiologics during HTOs, there is at present no consensus if orthobiologics should be routinely used in HTOs. The aim of this study is to qualitatively evaluate the current evidence reporting outcomes of intra-articular injection of orthobiologics in patients undergoing HTO for OA of the knee.

## Methods

### Information sources and selection of studies

**Fig. 1:**
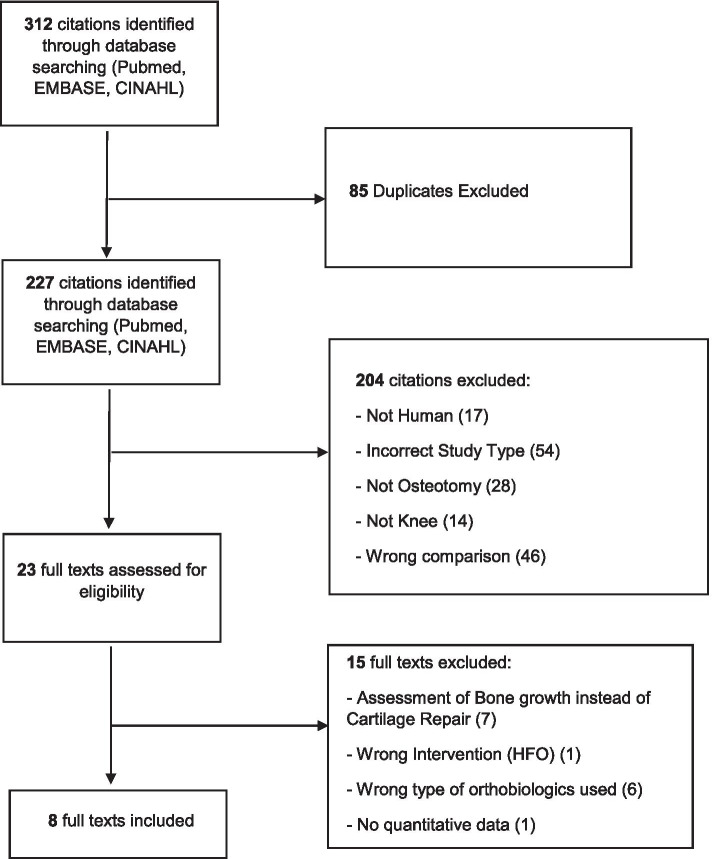
PRISMA Flowchart. The search workflow was performed in accordance to the Preferred Reporting Items for Systematic Reviews and Meta-Analyses (PRISMA)

An electronic search was performed by two independent authors (B.B. and A.H.) in the PUBMED, EMBASE, and CINAHL databases to identify all relevant studies published up to 10 July 2021. The search string used to query citation titles and abstracts was as follows: (Knee) AND (Osteotomy) AND (Biologics OR blood products OR PRP OR BMAC OR MSC OR Orthobiologics OR (Adipose derived OR Adipose derived mesenchymal stem cell OR synovial mesenchymal stem cell OR bone marrow mesenchymal stem cell) OR hUCB OR allogenic products OR amniotic fluid OR autologous conditioned serum OR stromal vascular fraction OR microfragmented adipose tissue OR PRGF OR amniotic membrane)”. This review was not registered on the PROSPERO database. The search workflow was in adherence to the Preferred Reporting Items for Systematic Reviews and Meta-Analyses (PRISMA) [16], and is showcased in Fig. 1.

To identify studies to be included in the final review, the articles were independently assessed by two authors, B.B. and A.H., to determine eligibility for inclusion in the analysis. Any disagreements were resolved by consensus discussion among the authors. A total of eight studies were included in the final review.

### Eligibility criteria

We included clinical trials of any level of evidence, reporting outcomes following HTO and concurrent injection of orthobiologics, including mesenchymal stem cells (MSCs),platelet-rich plasma (PRP), plasma rich in growth factors (PRGF), amniotic products, adipose-derived products, bone marrow aspirate concentrate (BMAC) or autologous conditioned serum with a minimum number of 10 patients treated. Case reports, review articles, published abstracts, studies involving less than 10 patients, and duplicate data (the most recent series was included) were excluded from this review. Studies which evaluated only implanted orthobiologics (including implanted MSCs) were excluded because they are considered reparative procedures and outcomes would be expected to be significantly different as compared to injected orthobiologics. Studies which compared implanted with injected orthobiologics were included for their data on the injected orthobiologics. Articles not written in English, or where access to the full text was unavailable, were also excluded.

### Data collection and statistical analysis

A total of 312 records were identified, of which 227 remained after removal of duplicates. Following Title and Abstract Screening, 23 Articles were identified and assessed in full text screening. Seven articles were then excluded because of their assessment of bone growth rather than cartilage repair, with a further eight articles excluded due to high fibular osteotomy (HFO) procedure instead of HTO, implantation of MSCs and lack of quantitative data.

All data from the texts, figures, and tables of the included studies were extracted to Microsoft Excel spreadsheet software for analysis and review. The specific information extracted included the following: (1) study details, including study design and level of evidence, (2) study population details, including number of patients, the size of the control group (if any), and the surgical procedures performed, (3) objective of study (4) intervention instituted, (5) Biologics system used and composition and quality of PRP (if PRP was used) (6) outcomes studied and criteria/scores used to quantify them and (7) results and any reported complications.

### Quality assessment of studies

The quality of the Randomised Controlled Trials (RCT) included in this study was assessed using the Cochrane Collaboration risk assessment tool [17] while non-randomized studies were assessed using the Risk of Bias in Non-Randomised Studies – of Intervention (ROBINS-I) tool [18]. The results of the Quality Assessment are detailed in Table 1.

**Table 1:** Risk of bias in included studies

**RCTs**	**Random Sequence Generation**	**Allocation Concealment**	**Blinding of participants and personnel**	**Blinding of outcome assessment**	**Incomplete outcome data**	**Selective reporting**	**Other Bias**	
D’Elia *et al*, Revista Brasileira de Ortopedia 2015 [19]	Low Risk	Low Risk	Unclear Risk	Unclear Risk	Low Risk	Unclear Risk	Unclear Risk	
Wong *et al*, Arthroscopy 2013 [20]	Low Risk	Low Risk	Low Risk	Unclear Risk	Low Risk	Low Risk	Unclear Risk	
Koh *et al*, Arthroscopy 2014 [21]	Low Risk	Low Risk	Low Risk	Unclear Risk	Low Risk	Low Risk	High Risk	
**Non- RCTs**	**Confounding**	**Selection of Participants**	**Classification of interventions**	**Deviations from intended interventions**	**Missing Data**	**Measurement of outcomes**	**Selection of reported results**	**Overall ROB judgements**
Magnanelli *et al*, Acta Biomedica 2020 [22]	Moderate	Moderate	Low	Low	Low	Low	Low	Moderate
Kim *et al*, American Journal of Sports Medicine 2018 [23]	Moderate	Moderate	Low	Low	Low	Low	Low	Moderate
Lee *et al*, Arthroscopy: The Journal of Arthroscopic and Related Surgery 2021 [24]	Moderate	Moderate	Low	Low	Low	Low	Low	Moderate
Jin *et al*, Knee Surgery, Sports Traumatology, Arthroscopy 2021 [15]	Moderate	Moderate	Low	Low	Low	Low	Low	Moderate
Yang *et al*, Knee Surgery, Sports Traumatology, Arthroscopy 2021 [25]	Moderate	Low	Low	Low	Low	Low	Low	Moderate

## Results

The eight studies [15, 19–25] included in this systematic review included a total of 585 patients. The results are presented according to the utilised orthobiologic agent as follows: two studies evaluated PRP, four evaluated injected culture-expanded MSCs, and four evaluated BMAC which were point-of-care unexpanded MSCs. Two studies included the use of dual orthobiologic agents [19, 21]. For studies with patients that underwent second-look arthroscopy, these were conducted within a range of 1 to 2 years following index surgery. All other data was collected within a range of one to three-and-a-half years post-procedure. With regards to study design, three (37.5%) studies provided Level II evidence and five (62.5%) studies provided Level III evidence. Characteristics of the studies are summarized in Table 2.

**Table 2:** Summary of included studies

**Study**	**Level of Evidence**	**Type of Osteotomy Performed**	**Intervention**	**Number of Patients in Intervention Group**	**Number of Patients in Control Group**	**Complications**
**D’Elia *et al*, Revista Brasileira de Ortopedia 2015** [19]	II	Opening Wedge HTO	PRP with BMAC	11	14	Nil reported
**Lee *et al*, Arthroscopy: The Journal of Arthroscopic and Related Surgery 2021** [24]	III	HTO	Microfracture with BMAC (42 patients) Microfracture with hUCB-MSC (32 patients)	74	N.A	Nil reported
**Jin *et al*, Knee Surgery, Sports Traumatology, Arthroscopy 2021** [15]	III	HTO	Microfracture with BMAC	48	43	Nil reported
**Yang *et al*, Knee Surgery, Sports Traumatology, Arthroscopy 2021** [25]	III	HTO	BMAC (55 Patients) hUCB-MSCs (55 Patients)	110	N.A	BMAC: one patient complained of postoperative stiffness
**Kim *et al*, American Journal of Sports Medicine 2018** [23]	III	HTO	MSCs	50	50	Nil reported
**Magnanelli *et al*, Acta Biomedica 2020** [22]	III	HTO	Autologous adipose derived stem cells	42	43	Nil reported
**Koh *et al*, Arthroscopy 2014** [21]	II	Opening Wedge HTO	PRP with MSCs	21	23	Nil reported
**Wong *et al*, Arthroscopy 2013** [20]	II	Medial Opening Wedge HTO	Cultured MSCs with Hyaluronic Acid	28	28	Nil reported

*HTO* High Tibial Osteotomy, *PRP* Platelet-Rich Plasma, *MSCs* Mesenchymal Stem Cells, *hUCB-MSCs* Human Umbilical Cord Blood-Derived Mesenchymal Stem Cells, *BMAC* Bone Marrow Aspirate Concentrate

### Scoring systems utilized

Multiple evaluation tools were utilized in the eight studies. The criteria, grading and descriptions of the systems discussed are listed here.

**The ICRS-CRA score** [26] has three components of evaluation: (i) degree of defect repair, (ii) integration to border zone, and (iii) macroscopic appearance. These components are graded normal (Grade I), nearly normal (Grade II), abnormal (Grade III), and severely abnormal (Grade IV). All studies reported second-look arthroscopy being conducted at a minimum of 1 year duration post-operatively. Four of the eight studies [15, 23–25] utilised this system.

**The Koshino Staging System** evaluates the status of the regenerated cartilage according to the macroscopic staging system described by Koshino et al. [9]. The staging system grades the regenerated cartilage as follows: (i) no regenerative change (Stage A), (ii) pink fibrous tissue with or without partial coverage with white fibrocartilage (Stage B), (iii) total cartilage regeneration with white overgrown cartilage (Stage C-1), and (iv) total cartilage regeneration with white even smooth cartilage (Stage C-2). All studies reported second-look arthroscopy being conducted at a minimum of 1-year following index surgery. Two of the eight [15, 25] studies utilised this system.

**The International Knee Documentation Committee (IKDC) Questionnaire** [8] is a subjective scale that provides patients with an overall function score. Consisting of three categories, (i) symptoms, (ii) sports activity, and (iii) knee function, it provides a means of assessing postoperative clinical and functional outcomes of procedures on the knee. Irrgang et al. [27] previously reported that the Minimum Clinically Important Difference (MCID) for IKDC following cartilage restoration procedures was 9.8. This was met by the five studies that reported IKDC as an outcome [15, 20, 22, 23, 25].

**The Knee Injury and Osteoarthritis Outcome (KOOS) score** [28] is a subjective questionnaire that assesses long and short-term impact on the patient post knee injury. It consists of five categories (i) pain, (ii) symptoms, (iii) activities of daily living, (iv) sport and recreation function and (v) quality of life relating to the knee. It is used to assess the course of the knee injury and outcome of treatments. Three of eight studies [21, 22, 25] utilised this system.

**The Lysholm Knee Scoring System** [29] is a patient-reported system used to assess a patients’ knee-specific symptoms. It consists of eight categories (i) pain, (ii) instability, (iii) locking, (iv) swelling, (v) limp, (vi) stair climbing, (vii) squatting, and (viii) need for support. Four of eight studies [20–23] utilised this scoring system.

**The Western Ontario and McMaster Universities Arthritis (WOMAC) Index** [30] is a self-administered questionnaire used to assess OA in the hip or knee. It consists of three categories (i) pain, (ii) stiffness and (iii) physical function. The MCID for WOMAC has been reported to be 15.0 [24]. This was met by the two studies that utilized the WOMAC index as an outcome [15, 24].

**The Visual Analog Scale (VAS)** [31] is a subjective single-item scale used to evaluate the pain intensity experienced by the patient. Two of eight [19, 21] studies utilised this scale.

**The Tegner Activity Scale** [29] is a single-item scale used to assess level of activity based on work and sports pre and post injury. Three of eight studies [20, 22, 25] utilised this scale.

**The Knee Society Score (KSS)** [32] is used to assess the patients’ knee and functional outcomes before and after treatment. It consists of two categories, pain and function. The MCID for the KSS pain category and function scores has been reported to be 3.0 and 5.6 respectively [24]. These were met by the two studies that utilized the KSS pain and function scores as outcome measures [15, 24].

### PRP studies

Two studies [19, 21] evaluated PRP combined with high tibial osteotomy. The results of these studies are summarised in Table 3. D’Elia et al. [19] reported outcomes assessed with post-operative VAS in patients who underwent HTO with PRP and BMAC versus iliac bone graft. There was no significant difference between the groups (*p* = 0.538).

**Table 3:** Clinical outcomes of studies utilising platelet-rich plasma

**Study**	**Type of Osteotomy Performed**	**Intervention**	**Number of Patients in Intervention Group**	**Number of Patients in Control Group**	**Number of Patients undergoing second-look Arthroscopy**	**Pre-OP VAS Score**	**Post-OP VAS score**	**Pre-OP Kanamiya Grading**	**Post-Op Kanamiya Grading**	**Pre-OP Lysholm Score**	**Post-OP Lysholm Score**	**Pre-OP KOOS Score**	**Post-OP KOOS Score**
**D’Elia *et al*, Revista Brasileira de Ortopedia 2015** [19]	HTO	PRP with BMAC	11	14	N.A	Not Reported		Not Reported	Not Reported	Not Reported	Not Reported	Not Reported	Not Reported
**Koh *et al,* Arthroscopy 2014** [21]	HTO	PRP with Adipose- derived MSC	21	23	44/44 patients at mean 19.8 months post-op	PRP (control) (*n* = 23) v PRP-MSC (*n* = 21) 45.4 ± 7.1 v 44.3 ± 5.7		Not Reported	PRP (control) (*n* = 23) v PRP-MSC (*n* = 21) Grade I: 11 (47.8%) v 1 (4.8%) Grade II: 11 (47.8%) v 9 (42.9%) Grade III: 1 (4.3%) v 8 (38.1%) Grade IV: 0 (0%) v 3 (14.3%)	PRP (control) (*n* = 23) v PRP-MSC (*n* = 21) 56.7 ± 12.2 v 55.7 ± 11.5	PRP (control) (*n* = 23) v PRP-MSC (*n* = 21) At latest follow-up (mean 24.6 months (PRP) and 24.2 months (PRP-MSC)) 80.6 ± 13.5 v 84.7 ± 16.2	Not Reported	PRP (control) (*n* = 23) v PRP-MSC (*n* = 21) At latest follow-up (mean 24.6 months (PRP) and 24.2 months (PRP-MSC)): Pain subscale: 74.0 ± 5.7 v 81.2 ± 6.9 Symptom subscale: 75.4 ± 8.5 v 82.8 ± 7.2

*VAS* Visual Analogue Scale, *KOOS* Knee injury and Osteoarthritis Outcome Score, *HTO* High Tibial Osteotomy, *MSCs* Mesenchymal Stem Cells, *PRP* Platelet-Rich Plasma, *BMAC* Bone Marrow Aspirate Concentrate

Koh et al. [21] reported outcomes in patients who underwent HTO with injection of PRP and adipose-derived MSCs versus patients who underwent HTO with injection of PRP only. They reported the Lysholm score, VAS score and KOOS scoring system following surgery. There were no significant differences (*p* = 0.357) in the Lysholm score between the two groups. VAS score was significantly better in the group which received PRP in combination with adipose-derived MSCs (*p* < 0.001). Similarly, the KOOS pain subscale (*p* < 0.001) and symptoms subscale (*p* < 0.001) showed greater improvement in the group which received PRP in combination with adipose-derived MSCs.

### BMAC studies

Four studies evaluated BMAC used in combination with HTO [15, 19, 24, 25]. The results of these studies are summarised in Table 4. The results of D’Elia et al. [19] have been discussed in the PRP results section above.

**Table 4:** Clinical outcomes of studies utilising bone marrow aspirate concentrate

**Study**	**Type of Osteotomy Performed**	**Intervention**	**Number of Patients in Intervention Group**	**Number of Patients in Control Group**	**Number of Patients undergoing second-look Arthroscopy**	**Pre-OP Koshino Staging**	**Post-OP Koshino Staging**	**Pre-OP ICRS-CRA**	**Post-OP ICRS-CRA**	**Pre-OP IKDC Score**	**Post-OP IKDC Score**	**Pre-OP WOMAC Score**	**Post-OP WOMAC score**
**Jin *et al*, Knee Surgery, Sports Traumatology, Arthroscopy 2021** [15]	HTO	Microfracture with BMAC	48	43	64/91 at mean 2 years post-op	Not Reported	Group I (*n* = 31) vs Group II (*n* = 33): Regeneration Stage: Stage A: 5 (16.1%) v 2 (6.1%) Stage B: 16 (51.6%) v 15 (45.5%) Stage C-1: 9 (29.0%) v 14 (42.4%) Stage C-2: 1 (3.2%) v 2 (6.1%)	Group I (*n* = 43) v Group II (*n* = 48) Grade III: 38 v 41 Grade IV: 5 v 7	Group I (*n* = 31) vs Group II (*n* = 33) Grade I: 0 v 1 Grade II: 12 v 18 Grade III: 10 v 11	Group I (*n* = 43) v Group II (*n* = 48): 33.7 ± 9.4 vs 35.3 ± 12.6	Group I (*n* = 43) v Group II (*n* = 48): At 1 year: 67.0 ± 10.6 vs 71.3 ± 11.2	Group I (*n* = 43) vs II (*n* = 48) 47.5 ± 10.4 vs 46.9 ± 13.9	Group I (*n* = 43) vs II (*n* = 48) 20.4 ± 9.7 vs 16.3 ± 9.8
**Yang *et al*, Knee Surgery, Sports Traumatology, Arthroscopy 2021** [25]	HTO	BMAC (55 Patients) hUCB-MSCs (55 Patients)	110	N.A	81/110 at mean 17 months post-op	Not Reported	BMAC (*n* = 37) v hUCB-MSC (*n* = 44) Stage A: 4 (10.8%) v 0 (0%) Stage B: 12 (32.4%) v 12 (27.3%) Stage C: 21 (56.8%) v 32 (72.7%)	BMAC (*n* = 55) vs hUBC-MSC (*n* = 55) Grade III: 5v3 Grade IV: 50 v 52	BMAC (*n* = 37) v hUBC-MSC (*n* = 44) Grade I: 1 v 4 Grade II: 20 v 30 Grade III: 11 v 10 Grade IV: 5 v 0	BMAC (*n* = 55) v hUCB-MSC (*n* = 55) 36.2 ± 3.0 v 35.4 ± 5.5	BMAC (*n* = 55) v hUCB-MSC (*n* = 55) At latest follow-up (mean 33.0 months): 72.8 ± 5.8 v 73.3 ± 9.8	Not Reported	Not reported
**Lee *et al*, Arthroscopy: The Journal of Arthroscopic and Related Surgery 2021** [24]	HTO	BMAC (42 Patients) hUCB-MSCs (32 Patients)	74	N.A	74/74 after minimum 1 year post-op	Not Reported	Not reported	Not Reported	BMAC (*n* = 42 Patients) v hUCB-MSC (*n* = 32 Patients)I: 1 v 6 II: 18 v 20 III: 12 v 6 IV: 11 v 0	Not reported	Not Reported	BMAC (*n* = 42 Patients) v hUCB-MSC (*n* = 32 Patients)43.9 ± 12.7 v 45.2 ± 8.8	BMAC (*n* = 42 Patients) v hUCB-MSC (*n* = 32 Patients) At latest follow-up:23.4 ± 11.6 v 19.5 ± 15.58
**D’Elia *et al*, Revista Brasileira de Ortopedia 2015** [19]	HTO	PRP with BMAC	11	14	N.A	Not Reported	Not Reported	Not Reported	Not Reported	Not Reported	Not Reported	Not Reported	Not Reported

**Study**	**Pre-OP KSS Score**	**Post-OP KSS Score**	**Pre-OP KOOS Score**	**Post-OP KOOS Score**	**Pre-OP SF-36 Score**	**Post-OP SF-36 Score**	**Pre-OP Tegner Activity Scale**	**Post-OP Tegner Activity Scale**	**Pre-OP HSS Score**	**Post-OP HSS Score**	**Pre-OP VAS**	**Post-OP VAS**
**Jin *et al*, Knee Surgery, Sports Traumatology, Arthroscopy 2021** [15]	Group I (*n* = 43) vs II (*n* = 48) Pain Subscale: 27.0 ± 8.5 vs 27.2 ± 7.6 Function Subscale: 60.6 ± 11.0 vs 58.9 ± 13.3	Group I (*n* = 43) vs II (*n* = 48) At Final followup (Mean 22.8 months for Group I and 20.3 months for Group II) Pain Subscale: 39.7 ± 6.5 vs 42.6 ± 7.2 Function Subscale: 88.8 ± 8.2 vs 91.0 ± 10.2	Not Reported	Not Reported	Not Reported	Not Reported	Not Reported	Not Reported	Not Reported	Not Reported	Not Reported	Not Reported
**Yang *et al*, Knee Surgery, Sports Traumatology, Arthroscopy 2021** [25]	Not Reported	Not Reported	BMAC (*n* = 55) v hUBC (*n* = 55) 1)Pain: 42.3 ± 3.7 v 41.4 ± 6.5 2)Symptoms:40.9 ± 5.1 v 39.5 ± 6.9 3)ADL 52.0 ± 7.1 v 51.5 ± 8.4 4)Sports and rec 23.8 ± 7.0 v 23.7 ± 9.2 5)QOL 31.1 ± 4.8 v 29.8 ± 6.3	BMAC (*n* = 55) v hUBC (*n* = 55) At latest follow-up (mean 33.0 months): 1)Pain: 81.7 ± 6.4 v 83.1 ± 8.3 2)Symptoms: 79.2 ± 7.5 v 79.4 ± 8.8 3)ADL: 82.4 ± 5.0 v 83.1 ± 5.8 4)Sports and rec: 62.0 ± 11.9 v 63.2 ± 10.7 5)QOL: 72.4 ± 6.8 v 73.8 ± 8.7	BMAC (*n* = 55) v hUBC (*n* = 55) Physical Component: 42.2 ± 3.5 v 41.5 ± 5.5 Mental Component: 57.2 ± 8.0 v 57.0 ± 9.2	BMAC (*n* = 55) v hUBC (*n* = 55) At latest follow-up (mean 33.0 months) Physical Component: 64.7 ± 5.9 v 65.4 ± 7.9Mental Component: 64.0 ± 8.7 v 64.7 ± 8.8	BMAC (*n* = 55) v hUBC (*n* = 55) 2.3 ± 0.9 v 2.2 ± 0.8	BMAC (*n* = 55) v hUBC (*n* = 55) At latest follow-up (mean 33.0 months) 4.0 ± 0.5 v 4.1 ± 0.5	Not Reported	Not Reported	Not Reported	Not Reported
**Lee *et al*, Arthroscopy: The Journal of Arthroscopic and Related Surgery 2021** [24]	BMAC (*n* = 42 Patients) v hUCB-MSC (*n* = 32 Patients) Pain Subscale: 30.8 ± 11.0 v 31.6 ± 10.4 Function Subscale: 62.3 ± 11.9 v 63.1 ± 11.2	BMAC (*n* = 42 Patients) v hUCB-MSC (*n* = 32 Patients) At latest follow-up Pain Subscale: 40.6 ± 9.1 v 42.8 ± 7.9 Function Subscale: 80.1 ± 15.0 v 82.4 ± 15.5	Not Reported	Not Reported	Not Reported	Not Reported	Not Reported	Not Reported	BMAC (*n* = 42 Patients) v hUCB-MSC (*n* = 32 Patients) 52.9 ± 12.9 v 56.1 ± 10.6	BMAC (*n* = 42 Patients) v hUCB-MSC (*n* = 32 Patients) At latest follow-up 79.2 ± 11.5 v 84.6 ± 15.5	Not Reported	Not Reported
**D’Elia *et al*, Revista Brasileira de Ortopedia 2015** [19]	Not Reported	Not Reported	Not Reported	Not Reported	Not Reported	Not Reported	Not Reported	Not Reported	Not Reported	Not Reported	Not Reported	Control (*n* = 14) v PRP-BMAC (*n* = 11) 24 h post-op: 5.1 ± 2.9 v 4.4 ± 2.7

*ICRS-CRA* International Cartilage Repair Society – Cartilage Assessment, *IKDC* International Knee Documentation Committee, *WOMAC* Western Ontario and McMaster Universities Arthritis Index, *KSS* Knee Society Score, *KOOS* Knee injury and Osteoarthritis Outcome Score, *SF-36* Short Form 36, *HSS* Hospital for Special Surgery, *VAS* Visual Analogue Scale, *HTO* High Tibial Osteotomy, *BMAC* Bone Marrow Aspirate Concentrate, *ADL* Activities of Daily Living, *QOL* Quality of Life, *hUCB-MSCs* Human Umbilical Cord Blood-Derived Mesenchymal Stem Cells

Jin et al. [15] reported outcomes in patients who underwent HTO with BMAC augmentation against a control group of patients who underwent HTO with microfracture (MFx) alone. The results in this study were reported using the following scoring systems, ICRS-CRA, Koshino Staging System, WOMAC Index, IKDC, and the KSS pain and function score. There was a statistically significant (*p* = 0.035) improvement in the mean ICRS-CRA grade of the group that had the BMAC augmentation versus the group that had MFx alone. There were no significant differences (*p* = 0.187) found between the two groups with regards to the Koshino Staging System score. There were also no significant differences between the two groups when assessed with the WOMAC Index (*p* = 0.297), IKDC (*p* = 0.260), KSS pain (*p* = 0.136) and function (*p* = 0.445).

Yang et al. [25] reported outcomes in patients who underwent HTO with BMAC versus HTO with human umbilical cord blood-derived MSCs (hUCB-MSC). The results in this study were reported using the following scoring systems, ICRS-CRA, Koshino Staging System, IKDC, KOOS, and the Tegner Activity Scale.

With regards to ICRS-CRA, Yang et al. [25] reported a statistically significant (*p* = 0.040) difference between the two groups. In their study, the BMAC group achieved significantly improved clinical and macroscopic outcomes, but worse macroscopic outcomes against a comparison group of patients who underwent hUCB-MSC implantation. Outcomes assessed with the Koshino Staging System showed significantly (*p* = 0.057) better cartilage regeneration in the group who underwent HTO with hUCB-MSC implantation, versus the group who underwent HTO with BMAC augmentation. There were no significant differences reported between the scores obtained by the two groups at the final follow up for the IKDC (*p* = 0.092), Tegner Activity Scale (*p* = 0.858) and KOOS (all subcategories *p* > 0.05).

Lee et al. [24] reported outcomes following HTO and MFx with BMAC versus HTO and MFx with hUCB-MSC. The results in this study were reported using the following scoring systems, ICRS-CRA, WOMAC index, KSS pain and function score. Lee et al. [24] corroborated the findings of Yang et al. [25] with regards to the ICRS-CRA score. The group that underwent BMAC augmentation showed significantly worse cartilage regeneration in both the medial femoral condyle (*p* = 0.001) and medial tibial condyle (*p* = 0.001) than the group that underwent hUCB-MSC implantation. There were no other significant differences between the two groups for the WOMAC Index (*p* = 0.080) and the KSS pain (*p* = 0.380) and function (*p* = 0.437) scores.

### Injected MSCs studies

Four studies [20–23] reported outcomes following HTO and injected MSCs. The results of these studies are summarized in Table 5. The results reported by Koh et al. [21] were discussed in the PRP results section above. In all these studies, there was culture expansion of the MSCs.

**Table 5:** Clinical outcomes of studies utilising injected mesenchymal stem cells

**Study**	**Type of Osteotomy Performed**	**Intervention**	**Number of Patients in Intervention Group**	**Number of Patients in Control Group**	**Number of Patients undergoing second-look Arthroscopy**	**Pre-OP Lysholm Score**	**Post-OP Lysholm Score**	**Pre-OP IKDC Score**	**Post-OP IKDC Score**	**Pre-OP Tegner Activity Scale**	**Post-OP Tegner Activity Scale**
**Kim *et al*, American Journal of Sports Medicine 2018** [23]	HTO	Adipose-derived MSCs	50	50	100/100 at mean 12.4 months (control) and 12.7 months (MSC)	Control (*n* = 50) v MSC (*n* = 50) 56.7 ± 12.2 v 55.7 ± 11.9	Control (*n* = 50) v MSC (*n* = 50) At final follow-up (mean 38.8 months (control) & 37.2 months (MSC)) 80.5 ± 15.2v 84.7 ± 16.1	Control (*n* = 50) v MSC (*n* = 50) 38.4 ± 9.2 v 36.5 ± 4.2	Control (*n* = 50) v MSC (*n* = 50) At final follow-up (mean 38.8 months (control) & 37.2 months (MSC)) 56.8 ± 14.7 v 64.8 ± 13.4	Not reported	Not reported
**Koh *et al*, Arthroscopy 2014** [21]	HTO	PRP with Adipose-derived MSCs	21	23	44/44 at mean 19.8 months post-op	PRP (control) (*n* = 23) v PRP-MSC (*n* = 21) 56.7 ± 12.2 v 55.7 ± 11.5	PRP (control) (*n* = 23) v PRP-MSC (*n* = 21) At latest follow-up (mean 24.6 months (PRP) and 24.2 months (PRP-MSC)) 80.6 ± 13.5 v 84.7 ± 16.2	Not Reported	Not Reported	Not Reported	Not Reported
**Wong *et al*, Arthroscopy 2013** [20]	HTO	Cultured Bone Marrow-Derived MSCs with Hyaluronic Acid	28	28	N.A	MSC (*n* = 28) v Control (HTO) (*n* = 28) 41.9 ± 19.2v 50.4 ± 23.0	MSC (*n* = 28) v Control (HTO) (*n* = 28) At latest follow-up (mean of 2 years) Added improvement of 7.61 (95% CI, 1.44 to 13.79; *P* = .016)for MSC group v Control	MSC (*n* = 28) v Control (HTO) (*n* = 28) 36.0 ± 13.7 v 33.9 ± 11.4	MSC (*n* = 28) v Control (HTO) (*n* = 28) At latest follow-up (mean of 2 years) Added improvement of 7.65 (95% CI, 3.04 to 12.26; *P* = .001) for MSC Group v Control	MSC (*n* = 28) v Control (HTO) (*n* = 28) 0–2: 15 v 16 3–5: 13 v 11 > 5: 0 v 1	MSC (*n* = 28) v Control (HTO) (*n* = 28) At latest follow-up (mean of 2 years) Added improvement of 0.64 (95% CI, 0.10 to 1.19; *P* = .021)for MSC Group v Control
**Magnanelli *et al*, Acta Biomedica 2020** [22]	HTO	Autologous Adipose-Derived MSCs	42	43	N.A	Not Reported	MSC (*n* = 42) v Control (HTO) (*n* = 43) At latest follow-up (mean of 1 year) No significant difference found between both groups (*P* > 0.05)	Not Reported	MSC (*n* = 42) v Control (HTO) (*n* = 43) At latest follow-up (mean of 1 year) No significant difference found between both groups (*P* > 0.05)	Not Reported	MSC (*n* = 42) v Control (HTO) (*n* = 43) At latest follow-up (mean of 1 year) No significant difference found between both groups (*P* > 0.05)

**Study**	**Pre-OP Kanamiya Grading**	**Post-OP Kanamiya Grading**	**Pre-OP ICRS-CRA**	**Post-OP ICRS-CRA**	**Pre-OP VAS Score**	**Post-OP VAS Score**	**Pre-OP MOCART Score**	**Post-OP MOCART Score**	**Pre-OP KOOS Score**	**Post-OP KOOS Score**
**Kim *et al*, American Journal of Sports Medicine 2018** [23]	Not Reported	Not reported	Not Reported	Control (*n* = 50) v MSC (*n* = 50) Femoral Condyle: Grade I: 2 v 4 Grade II: 6 v 13 Grade III: 26 v 20 Grade IV: 16 v 13 Tibial Plateau: Grade I: 3v 5 Grade II: 9 v 14 Grade III: 20 v 19 Grade IV: 18 v 12	Not reported	Not reported	Not reported	Not reported	Not reported	Not reported
**Koh *et al*, Arthroscopy 2014** [21]	Not Reported	PRP (Control) (*n* = 23) vs PRP with MSC (*n* = 21):	Not Reported	Not Reported	PRP (control) (*n* = 23) v PRP-MSC (*n* = 21) 45.4 ± 7.1 v 44.3 ± 5.7	PRP (control) (*n* = 23) v PRP-MSC (*n* = 21) At latest follow-up (mean 24.6 months (PRP) and 24.2 months (PRP-MSC)) 16.2 ± 4.6 v 10.2 ± 5.7	Not Reported	Not Reported	Not Reported	Not Reported
**Wong *et al*, Arthroscopy 2013** [20]	Not Reported	Not Reported	Not Reported	Not Reported	Not Reported	Not Reported	Not Reported	MSC (*n* = 28) v Control (HTO) (*n* = 28) At latest follow-up (mean of 2 years) 62.32 ± 17.56 v 43.21 ± 13.55	Not Reported	Not Reported
**Magnanelli *et al*, Acta Biomedica 2020** [22]	Not Reported	Not Reported	Not Reported	Not Reported	Not Reported	Not Reported	Not Reported	Not Reported	Not Reported	MSC (*n* = 42) v Control (HTO) (*n* = 43) At latest follow-up (mean of 1 year) No significant difference found between both groups (*P* > 0.05) in terms of ADL 1) Pain: no significant difference between both groups 2) Symptoms: no significant difference between both groups 3) ADL: *p* < 0.05 between both groups, with the MSC Group getting better results 4) Sports and rec: no significant difference between both groups 5) QOL: no significant difference between both groups

*IKDC* International Knee Documentation Committee, *ICRS-CRA* International Cartilage Repair Society – Cartilage Assessment, *VAS* Visual Analogue Scale, *MOCART* Magnetic Resonance Observation of Cartilage Repair Tissue, *KOOS* Knee injury and Osteoarthritis Outcome Score, *HTO* High Tibial Osteotomy, *BMAC* Bone Marrow Aspirate Concentrate, *PRP* Platelet-Rich Plasma, *MSCs* Mesenchymal Stem Cells, *ADL* Activities of Daily Living, *QOL* Quality of Life

Magnanelli et al. [22] evaluated the effect of adipose-derived MSCs with HTO and compared this to a control group that underwent HTO alone. The results in this study were reported using the following systems, KOOS, IKDC, Lysholm Scoring system, and Tegner Activity Scale. For the KOOS system, significant (*P* < 0.05) improvement was found with regards to the activities of daily living category for the group treated with adipose derived MSCs. No significant differences were found in other categories of the KOOS system. No significant differences were found when using the IKDC, Lysholm Scoring System and the Tegner Activity Scale.

Kim et al. [23] compared outcomes between patients who underwent HTO with adipose-derived MSCs with a control group of patients who underwent HTO alone. The results in this study were reported using the following systems, ICRS-CRA, IKDC, and Lysholm Scoring System. Unlike the results of Magnanelli et al. [22], Kim et al. [23] reported a statistically significant improvement in the mean ICRS-CRA grade of patients who underwent HTO with MSC injection with respect to cartilage regeneration at both the femoral condyle (*p* = 0.015) and the tibial plateau (*p* = 0.002). IKDC scores showed a significant (*p* = 0.049) difference in scores between the two groups, with the intervention group obtaining better scores at the final follow up post-operatively. There was also significant (*p* = 0.041) difference between the Lysholm scores between the two groups, with the group receiving adipose-derived MSCs obtaining better results.

Wong et al. [20] reported outcomes following HTO and injection of MSCs combined with hyaluronic acid versus HTO and injection of hyaluronic acid alone. The results in this study were reported using the following systems: IKDC, Lysholm Scoring system and Tegner Activity Scale. The authors reported a statistically better results in the group that underwent HTO and injection of MSCs combined with hyaluronic acid (*p* = 0.001) in terms of IKDC scores, supporting the findings of Kim et al. [23]. There was also significant differences (*p* = 0.016) between the two groups when using the Lysholm scoring system and the Tegner Activity Scale (*p* = 0.021) with the intervention group showing greater improvement than the control group, further supporting the findings of Kim et al. [23].

### Complications

Out of 585 patients, there were no reports of severe postoperative complications nor any severe adverse reactions such as deep infections or failure of prosthesis implants. However, Yang et al. [25] reported one patient in the intervention group who underwent HTO with BMAC that complained of postoperative stiffness which self-resolved without the need of any follow-up procedures.

## Discussion

This systematic review aimed to qualitatively evaluate the current evidence reporting outcomes of intra-articular injection of orthobiologics in patients undergoing HTO for OA of the knee. The key finding reported in this study is that there is a significant improvement in cartilage repair and regeneration following HTO when a concomitant injected orthobiologic product is used, except in studies when the injected orthobiologic is compared to an intervention utilising implanted MSC such as in the studies conducted by Yang et al. [25] and Lee et al. [24]. In our systematic review, we excluded implanted MSCs due to the nature of the procedure being reparative as compared to injected orthobiologics which are considered regenerative procedures. Thus, it is only fair that implanted MSCs and other reparative procedures be evaluated separately from injected orthobiologics as it would be expected that reparative procedures lead to far better macroscopic outcomes. Regardless, the absolute outcomes reported by Lee et al. [24] and Yang et al. [25] regarding injected MSCs remained acceptable when compared to other studies in this review. However, the authors do report discordance between macroscopic outcomes (ICRS-CRA, Koshino) and clinical findings (IKDC, KOOS, Lysholm, WOMAC, VAS, Tegner, KSS). Furthermore, due to the lack of high-level evidence, differing follow-up schedules, heterogeneity of intervention procedures between studies, and lack of a cost–benefit analysis, it is difficult to ascertain the true benefit that the various orthobiologic modalities provide when used concurrently with HTO. Studies with longer term follow-up are required to analyse if the increased quality of the repaired cartilage translates to functional and quality of life (QoL) improvements. Nonetheless based on our review, all the orthobiologics utilised in intervention groups have demonstrated good safety profiles and improvement in outcomes of cartilage repair. Hence, there is promise and potential for orthobiologics being used as an effective concomitant option for surgeons performing HTO [33].

Orthobiologic agents are believed to inhibit inflammatory processes and promote tissue healing [34]. Based on our results, all three agents such as PRP, BMAC and MSCs have largely been successful in improving outcomes following concomitant use with HTO. However, differences exist between the various orthobiologic agents based on the outcome measures, and the time frame within which the data was gathered. With regards to macroscopic outcomes, none of the papers that evaluated PRP presented data using ICRS-CRA or Koshino staging. Among the included studies reporting data on injected MSCs, Kim et al. [23] was the only study that reported ICRS-CRA, with significant improved outcomes in the intervention group, in line with significant clinical outcomes according to IKDC and Lysholm scoring. In contrast, BMAC studies present a mismatch between macroscopic and clinical outcomes, with three studies [15, 24, 25] reporting significant macroscopic but insignificant clinical outcomes. This can be attributed to high levels of heterogeneity between the papers which evaluated BMAC. Further minor reasons for this mismatch include differing MSC sources, different study designs with different interventions, and difference in follow-up times.

Based on the clinical outcome scores reported by Koh et al. [21], Kim et al. [23] and Wong et al. [20], the use of injected MSCs combined with another orthobiologic agent such as PRP or used on its own in a HTO procedure tends to produce a significantly better outcome in terms of cartilage regeneration and pain reduction if compared to HTO alone or if another orthobiologic agent was used on its own. MSCs are able to differentiate into chondrocytes as well as produce extracellular matrix molecules that are vital in cartilage regeneration and maintenance [35]. Thus the use of injected MSCs alongside other orthobiologics such as PRP tends to increase its efficacy due to its potential to promote the proliferation of MSCs as well as help to increase the ECM production [35], possibly contributing to the better outcomes as discussed above.

The study by Wong et al. [20] was the only one which presented data according to the Magnetic Resonance Observation of Cartilage Repair Tissue (MOCART) Knee Score [36], reporting significantly improved cartilage coverage of lesions with the usage of MSCs in HTO versus the control group of HTO with Hyaluronic Acid. This was accompanied by significantly better integration of the regenerated cartilage to the border zone with a lower rate of visible defects, with an age-adjusted mean difference in total MOCART score of 19.6. Despite this being the only included paper which presented MRI-backed data with regards to cartilage regeneration, the authors believe that the findings are significant given that MOCART is an objective score that provides a standardised, reproducible, and semiquantitative approach for the morphological assessment of cartilage repair [37]. Further studies which present MRI-backed data such as MOCART would be useful to discuss the balance between mechanics and biology in the pathogenesis and treatment of Knee OA.

In addition to knee-specific and joint-specific outcomes, another potential benefit of orthobiologics in HTO could be the reduction of postoperative blood loss, which remains a major complication of knee surgery. Perioperative and post-operative bleeding has been found to be associated with tourniquet use [12], alongside other bleeding risks involved in surgery. D’Elia et al. [19] reported on the change in haemoglobin (Hb) and haematocrit (Hct) levels to evaluate the extent of blood loss 24 h postoperatively. No significant differences in the change in Hb and Hct levels (*p* = 0.820 and *p* = 0.323 respectively) pre- and postoperatively were reported. In current literature, several studies have reported the efficacy of PRP in reducing perioperative and postoperative bleeding. PRPs contain a high concentration of growth factors, thromboxane A2 and thrombin which would theoretically lead to more efficient platelet plug formation and haemostasis [38]. A meta-analysis done by Ma et al. [39] found that the use of PRP during total knee arthroplasty (TKA) significantly reduced total blood loss (*p* = 0.0005) and decreased Hb drop at post-operative day 1 (*p* = 0.008) when compared against a control group. Everts et al. [40] also reported similar results where the decline in Hb levels post-operative days one and two were significantly lower in the PRP group when compared against a control group (*p* < 0.001 and *p* < 0.01 respectively). Therefore, PRP seems to exhibit a procoagulant effect, or at the very least may have a role in reducing perioperative and postoperative blood loss. However, due to conflicting findings and lack of high-level evidence, further high-level trials which also include relevant parameters such as prothrombin time are required to evaluate the efficacy of PRPs and other orthobiologics in reducing blood loss.

Finally, OA is a heterogeneous and multifactorial pathology and the underlying mechanisms causing the disease might differ between patients [41]. Given that HTO is indicated primarily in moderately active, high-demand, and relatively younger patients [42], the rate of conversion to TKA in these patients undergoing HTO with orthobiologics is a pertinent area of future research. The current literature is understandably limited in this area, given the relatively new status of orthobiologics as a concurrent treatment modality in HTO.

### Strengths and limitations

In our search of the literature, Harris et al. [14] presented the only prior systematic review which explored the clinical outcomes of biologics on HTO. However, this analysis was based on the concomitant utilisation of articular cartilage surgery and/or meniscal allograft transplantation rather than orthobiologics. This current study is the first systematic review which attempts to evaluate clinical and macroscopic outcomes following HTO with concomitant use of orthobiologics. It adds to the literature by showing that patients achieved statistically significant improvement in outcomes following HTO with PRP, BMAC or injected MSCs. The heterogeneity of studies included in this review alludes to the fact that there is a need for more robust clinical trials with repeatable study designs across the spectrum of orthobiologics.

However, the findings discussed in this systematic review should be carefully considered in light of our limitations. Firstly, multiple studies lacked a comparison against a suitable control, thus the data was deemed insufficient for a meta-analysis to be carried out. Studies utilised different systems to assess cartilage healing and regeneration, resulting in the lack of a singular basis of comparison. Furthermore, significant improvements in cartilage healing and regeneration may not completely correlate to improvements in clinical and functional outcomes of the knee. This is pertinent given the known dissociation between radiographic signs and clinical symptoms in patients with osteoarthritis of the knee [43]. Despite some studies indicating the significant correlation between cartilage regeneration and clinical outcome [15, 19–21, 23, 25, 44–47], more robust clinical trials are required to assess the degree to which this correlation can be established, in order to provide a holistic evaluation of the desired levels of cartilage regeneration that are associated with improvements in patient quality of life. An accurate assessment of financial costs of the multiple treatment regimes would also be required for a reliable cost–benefit analysis.

### Future research direction

Based on our findings, there is a lack of high-level studies evaluating the effects of orthobiologic injections in conjunction with HTO. We hope that this systematic review will help lead the discussion, and encourage researchers to conduct more robust Level I and II clinical and translational studies. These would address factors and outcomes not discussed in this review such as, but not limited to, postoperative bleeding, cost–benefit analyses of treatment modalities, and other orthobiologic agents.

## Conclusion

Intra-articular injection of orthobiologics in patients undergoing HTO is safe and effective with good outcomes reported. Due to the lack of high level of evidence, further research is required before this can be considered standard of care.

## Data Availability

Not applicable.

## Abbreviations

HTO: High tibial osteotomy
BMAC: Bone marrow aspirate concentrate
PRP: Platelet-rich plasma
MSCs: Mesenchymal stem cells
MFx: Microfracture
IKDC: International Knee Documentation Committee
MCID: Minimum Clinically Important Difference
ICRS-CRA: International Cartilage Repair Society – Cartilage Assessment
VAS: Visual Analogue Scale
WOMAC: Western Ontario and McMaster Universities Arthritis Index
KSS: Knee Society Score
MOCART: Magnetic Resonance Observation of Cartilage Repair Tissue
KOOS: Knee injury and Osteoarthritis Outcome Score
SF-36: Short Form 36
ADL: Activities of daily living
QOL: Quality of life
